# Effect of surface removal following bleaching on the bond strength of enamel

**DOI:** 10.1186/s12903-019-0742-4

**Published:** 2019-03-27

**Authors:** Yi-ling Cheng, Joseph Musonda, Hui Cheng, Thomas Attin, Ming Zheng, Hao Yu

**Affiliations:** 1 Department of Prosthodontics, School and Hospital of Stomatology, Fujian Medical University, Fuzhou, China; 2 Clinic of Preventive Dentistry, Periodontology and Cariology, Center of Dental Medicine, University Zurich, Zurich, Switzerland; 3 Department of Applied Prosthodontics, Graduate School of Biomedical Sciences, Nagasaki University, Nagasaki, Japan

**Keywords:** Enamel, Hydrogen peroxide, Shear bond strength

## Abstract

**Background:**

A reduction in bond strength of bleached enamel has been confirmed in the literature. Although limited information is available, it is conceivable that the veneer preparation process may remove the impacted enamel and further eliminate the compromised bond strength between the composite resin and bleached enamel. This study aimed to evaluate the effect of surface removal following bleaching on the micro-shear bond strength (μSBS) of bleached enamel.

**Methods:**

Forty-eight specimens were prepared from bovine incisors and were randomly divided into 2 groups (*n* = 24): group B (bleaching with 40% hydrogen peroxide for 2 × 45 min with a 1-week interval) and group C (control group without bleaching treatment). Immediately after receiving the treatments, 0.5 mm of the enamel was removed from the specimen surface, followed by bonding of composite resin to the enamel surface. Each group was further divided into 2 subgroups of 12 specimens each: subgroup T (with 5000 thermocycles in water baths at 5 °C and 55 °C), and subgroup N (without thermocycling). The μSBS values were measured using a universal testing machine and subjected to two-way analysis of variance (α = 0.05). The fracture modes of the specimens were observed using a stereomicroscope.

**Results:**

The μSBS values of the different groups ranged from 21.42 to 25.21 MPa. Following a surface reduction of 0.5 mm, bleaching treatment and thermocycling did not significantly affect the μSBS values (*P* = 0.348 and *P* = 0.507, respectively). No significant interaction was found between the bleaching treatment and thermocycling (*P* = 0.514). All the groups exhibited a high percentage of mixed failures. Compared with group C, group B exhibited higher percentage of adhesive failure.

**Conclusion:**

The results suggested that the bonding procedure could be performed on the bleached enamel following a surface reduction of 0.5 mm immediately after the bleaching treatment.

## Background

Having whiter teeth has become increasingly popular in recent years because in some communities it has a direct effect on gaining individual confidence and social recognition [1]. Depending on the varied severity and types of tooth discoloration, tooth color can be improved by tooth bleaching, tooth scaling and polishing, and veneers, etc. [2].

Although tooth bleaching has been proven to be a safe and effective treatment [3, 4], it may present certain adverse effects on the enamel, such as surface morphological changes [5, 6], compositional changes [7, 8], alterations in the surface microhardness [9–11] and surface roughness [12]. Moreover, there is evidence that tooth bleaching may lead to reduction in bond strength of composite resin applied on previously bleached enamel [13, 14]. A reduction in bond strength of 25–60% has been reported when the bonding procedure is performed on an enamel surface immediately after bleaching [14–16]. During bleaching treatment, hydrogen peroxide undergoes ionic dissociation and increases the formation of free radicals, such as nascent oxygen and the hydroxyl radical, on the enamel surface [17]. Although the mechanism is still not clear, the reduced bond strength of bleached enamel has been related to the presence of residual free radicals due to the breakdown of hydrogen peroxide [14, 18] and alterations in the enamel composition and structure [6, 19] following the bleaching treatment. The residual oxygen in the interprismatic spaces can hamper resin infiltration and inhibit resin polymerization [20]. Moreover, morphological and compositional changes (e.g., porosity, loss of enamel prismatic form, loss of calcium, and changes in organic substances) in the enamel may weaken the adhesive interface and compromise bond strength [21, 22]. Therefore, bonding procedures should not be performed immediately after bleaching treatment [23]. A waiting period of 1–3 weeks has been advocated by various researchers [21, 24, 25]. In addition to the delayed bonding procedure, the application of antioxidant agents (e.g., sodium ascorbate, sodium bicarbonate, and grape seed extract) [18, 26, 27] and laser irradiation [28, 29] have been proposed to restore the compromised bond strength of bleached enamel. By neutralizing residual free radicals [30] and promoting micro-retentions in the enamel surface [28], antioxidant agents and laser irradiation have been shown to reverse the reduced bond strength between the composite resin and bleached enamel. However, it is important to point out that most of the above-mentioned studies measured the bond strength without thermocycling [24–29]. Thermocycling is the in vitro process of subjecting a restoration and tooth to temperature limits similar to those experienced in the oral cavity [31]. It would be of interest to investigate the effects of thermocycling on the bond strength between the composite resins and the bleached enamel.

In cases of severely discolored dentition and the need for tooth shape corrections, esthetic reconstructions, such as laminate and composite veneers, are often required after the bleaching treatment. Given that most of the negative effects of bleaching have been found on the enamel surface [9], it is conceivable that the veneer preparation process may remove the impacted enamel and further eliminate the compromised bond strength between the composite resin and bleached enamel. However, few studies have addressed this issue.

Therefore, the aim of the present study was to evaluate the effect of surface removal following bleaching on the micro-shear bond strength (μSBS) between composite resin and bleached enamel immediately after bleaching treatment. The following null hypotheses were tested: (1) that the bleaching treatment would not affect the bond strength between the composite resin and the enamel when a surface reduction of 0.5 mm was performed immediately following bleaching; (2) that the bond strength between the composite resin and the enamel would be the same after thermocycling.

## Methods

The research protocol was reviewed and approved by the Research Ethics Committee of School and Hospital of Stomatology, Fujian Medical University (No. 2015-CX-31).

### Specimen preparation

Forty-eight specimens were prepared from freshly extracted bovine incisors. The bovine teeth were collected as anonymous by-products of regular slaughtering of the cattle for human food consumption. Enamel blocks (4 mm × 4 mm × 4 mm) were cut from the middle third of the buccal surfaces using a low speed diamond saw (Isomet, Buehler, Lake Bluff, IL, USA) under water cooling. The enamel blocks were further embedded in acrylic resin (ZiRan, Nissin, Hangzhou, Zhejiang, China) using a polyvinylchloride ring mold of a diameter of 10 mm. After ultrasonically cleaning in distilled water for 1 min, the specimens were randomly divided into 2 groups of 24 specimens each, according to the bleaching treatment performed.

In group B (bleaching group), the specimens were bleached with 40% hydrogen peroxide gel (Opalescence Boost 40%, Ultradent Product Inc., South Jordan, Utah, USA). Two bleaching sessions with a 1-week interval were performed in a wet chamber at 37 °C. For each session, bleaching gels were applied to the enamel surface at a thickness of 1 mm for 15 min and were renewed three times. After each bleaching session, the bleaching gels were wash away from the specimen surfaces with distilled water. During the bleaching intervals, the specimens were stored in artificial saliva at 37 °C. The artificial saliva was mixed according to the formulation described by Klimek et al. [32]. 500 ml of artificial saliva contained 0.001 g ascorbic acid, 0.015 g glucose, 0.290 g NaCl, 0.085 g CaCl_2_, 0.080 g NH_4_Cl, 0.635 g KCl, 0.080 g NaSCN, 0.165 g KH_2_PO_4_, 0.100 g carbamide, 1.350 g mucin and 0.170 g Na_2_PO_4_.

In group C (control group), no bleaching treatment was performed on the specimen surfaces. The specimens were immersed in artificial saliva during the experimental period.

### Bonding procedure

Immediately following the abovementioned treatments, 0.5 mm of the enamel was removed from the specimen surface with the aid of a depth-cut diamond bur (DC .5, Premier Dental Products, Plymouth Meeting, PA, USA) and a series of carborundum discs (400#, 600#, Buehler), under water irrigation. Enamel reduction was controlled with a micrometer (Mitutoyo, Kyoto, Japan). The specimens were then ultrasonically cleaned in distilled water for 5 min to remove any debris. The surfaces were observed with a stereomicroscope (MM400, Nikon, Tokyo, Japan), and the specimens exposing the cracks or dentin surface were discarded.

After cleaning with pumice, the enamel surface of each specimen was etched with 35% phosphoric acid (Scotchbond Etchant, 3 M ESPE, St Paul, MN, USA) for 15 s, rinsed with distilled water for 20 s, and dried thoroughly. Two consecutive coats of adhesive agents (Adper Single Bond 2, 3 M ESPE, St Paul, MN, USA) were applied and light polymerized for 10 s with an LED light-curing unit (Elipar S10, 3 M ESPE, St Paul, MN, USA). A polyethylene tube with an internal diameter of 1 mm and a height of 1.5 mm was mounted on the enamel surface and filled with the composite resin (RelyX Veneer, 3 M ESPE, St Paul, MN, USA). The composite resin was then light cured using the abovementioned light-curing unit for 40 s. The excess material surrounding the tube was removed with a sharp scalpel. The specimens were stored at room temperature for 1 h prior to removal of the polyethylene tubes. All the specimens were then examined with a stereomicroscope to exclude any specimens with air bubbles, evident interfacial gaps, or any other bonding defects.

### Thermocycling

Each group was further divided into 2 subgroups (*n* = 12), according to the thermocycling procedure used.

In subgroup T, the specimens were thermocycled using a thermocycling machine (TC-501F, Weier, Tianjin, China) and then subjected to the μSBS test. Thermocycling was performed for 5000 cycles at bath temperatures of 5 °C and 55 °C, with a dwell time of 30 s in each bath and a transfer time of 10 s.

In subgroup N, the specimens were stored in distilled water at 37 °C for 24 h before undergoing the μSBS test.

### μSBS test

The μSBS test was performed using a universal testing machine (AGS-X, Shimadazu, Tokyo, Japan) with a cross-head speed of 1.0 mm/min until failure occurred. The maximum failure load (in N) was recorded and converted into MPa by dividing the failure load by the bonding area (0.785 mm^2^) [33].

After the test, the fracture surfaces were observed with a stereomicroscope under × 40 magnification. The failure modes were classified into 3 types: 1) adhesive failure: failure between the adhesive and the enamel or between the composite resin and the enamel; 2) cohesive failure: failure within the enamel or within the composite resin; and 3) mixed failure: fractures involving adhesive and cohesive failures simultaneously [20].

### Statistical analysis

The data were analyzed using SPSS software (version 20.0 for Windows, SPSS, Chicago, IL, USA) at α = 0.05. The assumption of normality was confirmed using the Kolmogorov-Smirnov test, and the equality of variances was confirmed with the Levene test. Two-way analysis of variances (ANOVA) was used to evaluate the effects of both the bleaching treatment and the thermocycling process and their interactions on the μSBS.

## Results

Table 1 presents the means and standard deviations of the μSBS values for the different groups. The group CN (unbleached specimens without thermocycling) showed the highest μSBS value (25.21 ± 5.53 MPa), while the group BT (bleached specimens with thermocycling) exhibited the lowest μSBS value (21.41 ± 7.22 MPa). Two-way ANOVA indicated no significant effects of either the bleaching treatment or thermocycling as well as no significant interaction between the two factors (Table 2).

**Table 1:** Means and standard deviations of the μSBS values (MPa) for different groups

Group	Subgroup	μSBS
B (bleaching)	T (with thermocycling)	21.41 (7.22)
	N (without thermocycling)	21.45 (8.87)
C (control)	T (with thermocycling)	22.10 (6.05)
	N (without thermocycling)	25.21 (5.53)

**Table 2:** Two-way ANOVA results for the μSBS values

Source of variance	Type III sum of squares	*df*	Mean square	*F*	*P*
Bleaching	49.146	1	49.146	0.906	0.348
Thermocycling	24.371	1	24.371	0.449	0.507
Bleaching x thermocycling	23.621	1	23.621	0.435	0.514
Error	1953.251	36	54.257		

**Fig. 1:**
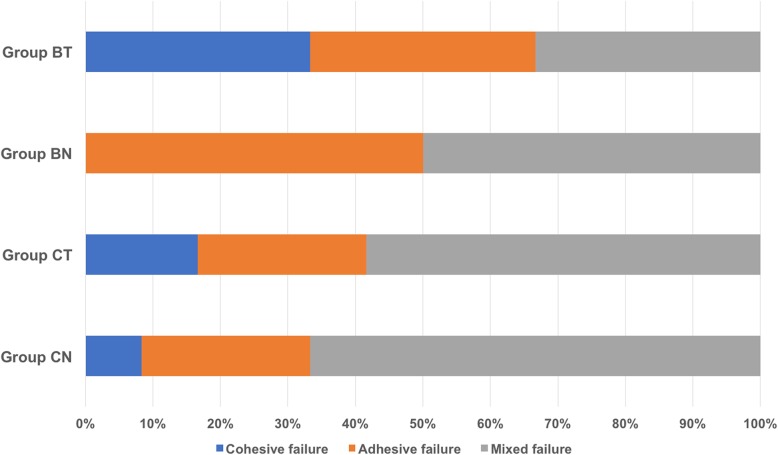
Distribution of failure modes among different groups

The frequencies of different failure modes are shown in Fig. 1. Mixed failure was the most prevalent failure mode, followed by adhesive failure and cohesive failure. Compared with group C, group B exhibited higher percentage of adhesive failure.

## Discussion

Based on the current results, the null hypotheses that the bleaching treatment would not affect the bond strength between the composite resins and the enamel when the surface is reduced by 0.5 mm immediately after bleaching and that the bond strength between the composite resin and the enamel remains the same after thermocycling were accepted.

The μSBS test was used to measure the bond strength of bleached enamel and to avoid the non-uniform distribution of interfacial stress that occurs during the macro-shear bond tests [21, 33]. Weerasinghe et al. [33] reported a μSBS value for grounded enamel of ~ 25 MPa, which is consistent with the present finding. The bond strength and failure mode differed between the aged and non-aged samples. The aged samples exhibited lower μSBS values compared with the non-aged samples, although the differences were not statistically significant. Temperature changes during the thermocycling process may amplify the coefficient of thermal expansion mismatch of the bonded materials, which generates mechanical stresses at the bonded interface, resulting in bonding degradation [34].

Veneers and other restorative procedures are often required after the bleaching treatment. Given that very limited information is available, the present study aimed to compare the bond strength of bleached and unbleached enamel after a surface removal of 0.5 mm, which was performed to simulate the tooth preparation process for laminate or composite veneers [33, 35]. After removing 0.5 mm of the superficial enamel, the specimens from the bleaching group exhibited similar μSBS values to those from the control group, regardless of thermocycling. Although the bond strength of bleached enamel without surface reduction was not tested in the present study, a reduction in the enamel bond strength after bleaching has been well documented by previous studies [14–16]. Evidence has also shown a reduction in resin tags as well as a lower degree of adhesive penetration into the bleached enamel substrate [13, 36]. The abovementioned phenomena have been attributed to the residual free radicals being released from the bleaching agents and to bleaching-induced structural changes in the enamel [14, 15, 25]. Although no information is available regarding the amount of residual free radicals or structural changes found at different depths of dental hard tissues, it is conceivable that changes due to bleaching occur primarily on the enamel surface. The residual oxygen and structural changes caused by bleaching would therefore be eliminated, at least to some extent, by the enamel reduction process. This hypothesis is supported by previous studies that found that the topical application of antioxidant agents [18, 26, 27] and the use of laser irritation [28, 29] could reverse the reduced bond strength of enamel after bleaching. Interestingly, although no significant differences in the bond strength were found, the mean bond strength of bleached enamel was lower than that for unbleached enamels, and the frequency of adhesive failure in the bleaching group was higher than that in the control group. These findings may indicate that the negative effects of bleaching were not completely reversed, even after the superficial layer of the enamel was removed. However, this hypothesis should be tested in future studies.

Due to morphological and physicochemical similarities, bovine teeth are a good alternative to human teeth for experimental procedures [37, 38]. Moreover, human and bovine teeth have been reported to exhibit similar behaviors during bleaching treatments [39]. Thus, the present study used bovine teeth as the testing substrates, in accordance with the previous studies [20, 28, 29, 40].

Based on the μSBS results, clinicians should be able to perform the bonding procedures using bleached enamel immediately after bleaching if 0.5 mm of the enamel is first removed from the tooth surface. The negative effects of bleaching on the bond strength of enamel have been suggested to be hydrogen-peroxide-concentration-dependent [20]. Because a 40% hydrogen peroxide gel was employed to simulate the worst-case scenario, the present findings may also be applicable to at-home bleaching, which uses a lower concentration of hydrogen peroxide or carbamide peroxide. However, further in vitro and in vivo studies are necessary to confirm this hypothesis.

## Conclusions

Within the limitations of the present study, it can be concluded that bleached and unbleached enamel exhibited similar μSBS values after removing 0.5 mm of the enamel from the surface.

## Abbreviations

ANOVA: analysis of variances
μSBS: micro-shear bond strength

## References

[1] Meireles SS, Goettems ML, Dantas RV, Bona AD, Santos IS, Demarco FF (2014). Changes in oral health related quality of life after dental bleaching in a double-blind randomized clinical trial. J Dent.

[2] Joiner A (2006). The bleaching of teeth: a review of the literature. J Dent.

[3] Meireles SS, Santos IS, Bona AD, Demarco FF (2010). A double-blind randomized clinical trial of two carbamide peroxide tooth bleaching agents: 2-year follow-up. J Dent.

[4] Knosel M, Attin R, Becker K, Attin T (2008). A randomized CIE L*a*b* evaluation of external bleaching therapy effects on fluorotic enamel stains. Quintessence Int.

[5] Al-Salehi SK, Wood DJ, Hatton PV (2007). The effect of 24h non-stop hydrogen peroxide concentration on bovine enamel and dentine mineral content and microhardness. J Dent.

[6] Grazioli G, Valente LL, Isolan CP, Pinheiro HA, Duarte CG, Munchow EA (2018). Bleaching and enamel surface interactions resulting from the use of highly-concentrated bleaching gels. Arch Oral Biol.

[7] Eimar H, Siciliano R, Abdallah MN, Nader SA, Amin WM, Martinez PP, Celemin A, Cerruti M, Tamimi F (2012). Hydrogen peroxide whitens teeth by oxidizing the organic structure. J Dent.

[8] Potocnik I, Kosec L, Gaspersic D (2000). Effect of 10% carbamide peroxide bleaching gel on enamel microhardness, microstructure, and mineral content. J Endod..

[9] Attin T, Schmidlin PR, Wegehaupt F, Wiegand A (2009). Influence of study design on the impact of bleaching agents on dental enamel microhardness: a review. Dent Mater.

[10] Attin T, Kocabiyik M, Buchalla W, Hannig C, Becker K (2003). Susceptibility of enamel surfaces to demineralization after application of fluoridated carbamide peroxide gels. Caries Res.

[11] Chen HP, Chang CH, Liu JK, Chuang SF, Yang JY (2008). Effect of fluoride containing bleaching agents on enamel surface properties. J Dent.

[12] Martin JM, de Almeida JB, Rosa EA, Soares P, Torno V, Rached RN, Mazur RF (2010). Effect of fluoride therapies on the surface roughness of human enamel exposed to bleaching agents. Quintessence Int.

[13] Briso AL, Toseto RM, Rahal V, dos Santos PH, Ambrosano GM (2012). Effect of sodium ascorbate on tag formation in bleached enamel. J Adhes Dent.

[14] Vidhya S, Srinivasulu S, Sujatha M, Mahalaxmi S (2011). Effect of grape seed extract on the bond strength of bleached enamel. Oper Dent.

[15] Lai SC, Tay FR, Cheung GS, Mak YF, Carvalho RM, Wei SH, Toledano M, Osorio R, Pashley DH (2002). Reversal of compromised bonding in bleached enamel. J Dent Res.

[16] Kunt GE, Yilmaz N, Sen S, Dede DO (2011). Effect of antioxidant treatment on the shear bond strength of composite resin to bleached enamel. Acta Odontol Scand.

[17] Minoux M, Serfaty R (2008). Vital tooth bleaching: biologic adverse effects-a review. Quintessence Int.

[18] Turkun M, Celik EU, Kaya AD, Arici M (2009). Can the hydrogel form of sodium ascorbate be used to reverse compromised bond strength after bleaching?. J Adhes Dent.

[19] Lima DA, Aguiar FH, Pini NI, Soares LE, Martin AA, Liporoni PC, Ambrosano GM, Lovadino JR (2015). In vitro effects of hydrogen peroxide combined with different activators for the in-office bleaching technique on enamel. Acta Odontol Scand.

[20] Guler E, Gonulol N, Ozyilmaz OY, Yucel AC (2013). Effect of sodium ascorbate on the bond strength of silorane and methacrylate composites after vital bleaching. Braz Oral Res.

[21] Bittencourt BF, Dominguez JA, Loguercio AD, Gomes JC, Gomes OM (2013). Influence of two different methods of delivering fluoride on bond strength and degree of conversion of an adhesive after bleaching. J Adhes Dent.

[22] Perdigao J, Francci C, Swift EJ, Ambrose WW, Lopes M (1998). Ultra-morphological study of the interaction of dental adhesives with carbamide peroxide-bleached enamel. Am J Dent.

[23] Yu H, Zhang C, Cheng S, Cheng H (2015). Effects of bleaching agents on dental restorative materials: a review of the literature and recommendation to dental practitioners and researchers. J Dent Sci.

[24] Cura M, Fuentes MV, Ceballos L (2015). Effect of low-concentration bleaching products on enamel bond strength at different elapsed times after bleaching treatment. Dent Mater J.

[25] Basting RT, Rodrigues JA, Serra MC, Pimenta LA (2004). Shear bond strength of enamel treated with seven carbamide peroxide bleaching agents. J Esthet Restor Dent.

[26] Alencar MS, Bombonatti JF, Maenosono RM, Soares AF, Wang L, Mondelli RF (2016). Effect of two antioxidants agents on microtensile bond strength to bleached enamel. Braz Dent J.

[27] Berger SB, De Souza Carreira RP, Guiraldo RD, Lopes MB, Pavan S, Giannini M, Bedran-Russo AK (2013). Can green tea be used to reverse compromised bond strength after bleaching?. Eur J Oral Sci.

[28] Lago AD, de Freitas PM, Netto NG (2011). Evaluation of the bond strength between a composite resin and enamel submitted to bleaching treatment and etched with Er:YAG laser. Photomed Laser Surg.

[29] Rocha Gomes Torres C, Caneppele TM, Del Moral de Lazari R, Ribeiro CF, Borges AB (2012). Effect of dental surface treatment with Nd:YAG and Er:YAG lasers on bond strength of resin composite to recently bleached enamel. Lasers Med Sci.

[30] da Cunha LF, Furuse AY, Mondelli RF, Mondelli J (2010). Compromised bond strength after root dentin deproteinization reversed with ascorbic acid. J Endod.

[31] Korkmaz Y, Gurgan S, Firat E, Nathanson D (2010). Effect of adhesives and thermocycling on the shear bond strength of a nano-composite to coronal and root dentin. Oper Dent.

[32] Klimek J, Hellwig E, Ahrens G (1982). Fluoride taken up by plaque, by the underlying enamel and by clean enamel from three fluoride compounds in vitro. Caries Res.

[33] Weerasinghe DS, Nikaido T, Wettasinghe KA, Abayakoon JB, Tagami J (2005). Micro-shear bond strength and morphological analysis of a self-etching primer adhesive system to fluorosed enamel. J Dent.

[34] Yu H, Yoshida K, Cheng H, Sawase T. Bonding of different self-adhesive resins to high-strength composite resin block treated with surface conditioning. J Prosthodont Res. 2019; 10.1016/j.jpor.2019.01.008. 10.1016/j.jpor.2019.01.008 30792147

[35] Burke FJ (2012). Survival rates for porcelain laminate veneers with special reference to the effect of preparation in dentin: a literature review. J Esthet Restor Dent.

[36] Wilson D, Xu C, Hong L, Wang Y (2009). Effects of different preparation procedures during tooth whitening on enamel bonding. J Mater Sci Mater Med.

[37] Laurance-Young P, Bozec L, Gracia L, Rees G, Lippert F, Lynch RJ, Knowles JC (2011). A review of the structure of human and bovine dental hard tissues and their physicochemical behaviour in relation to erosive challenge and remineralisation. J Dent.

[38] Camargo MA, Marques MM, de Cara AA (2008). Morphological analysis of human and bovine dentine by scanning electron microscope investigation. Arch Oral Biol.

[39] Attia ML, Aguiar FH, Mathias P, Ambrosano GM, Fontes CM, Liporoni PC (2009). The effect of coffee solution on tooth color during home bleaching applications. Am J Dent.

[40] Publio JD, D'Arce MB, Catelan A, Ambrosano GM, Aguiar FH, Lovadino JR, Lima DA (2016). Influence of enamel thickness on bleaching efficacy: an in-depth color analysis. Open Dent J.

